# The Mental Health Literacy and Prevention Survey (mHELP): a brief measure for assessing gatekeepers’ mental health literacy in prevention research

**DOI:** 10.3389/fpubh.2026.1868366

**Published:** 2026-08-18

**Authors:** Sarah Franke, Isabell Bendl, Sabrina Mittermeier, Carina Sauter, Arne Bürger

**Affiliations:** 1 Department of Child and Adolescent Psychiatry, Psychosomatics and Psychotherapy, University Hospital of Wuerzburg, Wuerzburg, Germany; 2 German Centre of Prevention Research in Mental Health, University of Wuerzburg, Wuerzburg, Germany

**Keywords:** gatekeeper, mental health, mental health literacy, prevention, validation

## Abstract

**Theoretical background:**

Mental health literacy (MHL) is a key target of prevention efforts to help gatekeepers such as teachers and other professionals recognize and respond to mental health problems in young people. To evaluate such mental health prevention initiatives, instruments that capture the multiple components of MHL in a concise and practical way are crucial. However, existing measures often assess only selected aspects of the construct or have not been comprehensively validated. Building on the established conceptualization of MHL, a brief and multidimensional measure suitable for prevention and implementation research is needed.

**Methods:**

The present study reports on the development and validation of the Mental Health Literacy and Prevention Survey (mHELP). Using baseline data from *N* = 556 teachers in Germany, we conducted confirmatory factor analyses (CFA) to examine the factor structure of the instrument. Moreover, internal consistency, test–retest reliability, split-half reliability, and construct validity were assessed.

**Results:**

Confirmatory factor analyses provided initial support for the proposed five-factor structure. Reliability was good for the total score as well as for the subscales Knowledge, Self-reported helping behavior, and Emotional understanding, whereas the Stigma and Confidence subscales showed weaker psychometric performance. Test–retest reliability over 6 months was moderate and split-half reliability of the total score was high. Initial evidence of construct validity was provided by expected associations with established measures of mental health knowledge and stigma, while low correlations with symptom measures suggested preliminary evidence of discriminant validity.

**Discussion:**

The mHELP represents a brief, multidimensional instrument for assessing MHL among gatekeepers, with the total score currently appearing to be the most robust indicator. At the subscale level, several dimensions showed satisfactory psychometric properties, whereas the Stigma and Confidence subscales require further refinement before separate interpretation can be recommended. By integrating multiple facets of MHL, including emotional understanding as a practice-informed component, the instrument may complement existing approaches while remaining practical for prevention research. Overall, the findings provide initial support for the utility of the mHELP, particularly at the total-score level, although further validation is needed, including refinement of selected subscales, sensitivity to change, and testing in more diverse and international samples.

## 1 Introduction

Mental health problems are among the most important social issues of recent decades. Indeed, the lifetime prevalence of any mental disorder is estimated to lie at 28–40% (1, 2), and 13–20% of all children and adolescents worldwide are reported to suffer from a mental health problem (3, 4). Beyond the associated individual burden such as reduced wellbeing, functional impairment, and diminished quality of life, mental disorders are linked to considerable economic costs for healthcare systems and society as a whole (5–7) due to direct expenditure for treatment and care, as well as indirect costs such as productivity losses, increased sickness absence, and early retirement (5–7). Importantly, half of all lifetime mental disorders emerge before the age of 15 years (2), underscoring the developmental nature of mental health problems and highlighting the critical importance of prevention and early intervention efforts. As such, addressing mental health risk factors at an early stage represents a key public health priority with the potential to prevent chronicity and reduce both individual suffering and long-term societal costs.

To counteract the growing individual and societal burden of mental disorders, there is an urgent need for prevention efforts that address mental health issues early on before they develop into chronic or severely impairing conditions. In the context of public health, *prevention* refers to interventions designed to reduce the occurrence, duration, or severity of disorders by addressing risk factors before, during, or after the onset of illness. A key framework for conceptualizing prevention strategies in mental health was provided by Mrazek & Haggerty (8), who distinguished between three main levels of prevention: *Universal prevention* targets entire populations, regardless of individual risk status, and aims to promote general health and prevent the onset of disorders at a population level. For example, written educational materials about healthy eating might be provided to all students in a school. *Selective prevention* focuses on subgroups of the population that have an elevated risk of developing a disorder due to specific biological, psychological, or social risk factors. For example, preventive intervention programs may be offered to children whose parents have a mental illness. *Indicated prevention* targets individuals who are already showing early or subclinical symptoms of a disorder but do not yet meet full diagnostic criteria. For example, brief cognitive-behavioral group interventions may be provided to adolescents who report elevated depressive symptoms but have not been formally diagnosed with depression.

In recent years many prevention programs have shifted their focus from directly targeting at-risk populations to training so-called *gatekeepers*. Gatekeepers are individuals who, due to their social, educational, or professional roles (e.g., parents, teachers or youth workers), have regular contact with members of the target population and are therefore well positioned to observe behavioral or emotional changes. Accordingly, prevention programs for gatekeepers typically aim to improve gatekeepers’ *mental health literacy* (MHL).

MHL is commonly understood as a multidimensional construct encompassing (1) knowledge about mental health and mental disorders, (2) attitudes and beliefs related to stigma, (3) confidence in one’s own ability to engage in appropriate help-seeking, and (4) self-reported behaviors, such as providing help to others experiencing mental health difficulties [based on (9–11)]. This multidimensional conceptualization reflects the assumption that factual knowledge alone is insufficient to promote preventive behavior and that attitudes, self-efficacy, and behavioral competencies play a central role in recognizing mental health problems and responding to them appropriately. In prevention-oriented gatekeeper contexts, participatory and practice-based perspectives may further highlight aspects that are not always explicitly represented in existing MHL measures, such as understanding emotional distress and the subjective experiences of individuals affected by mental health difficulties. These aspects may be particularly relevant for gatekeepers, as the sensitive recognition of mental health concerns and the provision of appropriate support often require not only factual knowledge, but also an understanding of how emotional distress may be experienced and expressed. Many existing prevention programs explicitly target improvements in participants’ MHL as a central intervention focus and a primary outcome measure of effectiveness [e.g., (12–14)]. Meta-analytic evidence suggests that such programs can have positive effects (15, 16), highlighting their potential to reduce risk factors and promote mental health at the population level. Nevertheless, findings need to be interpreted with caution due to substantial methodological heterogeneity across studies, including differences in theoretical foundations, target groups, intervention components, outcome measures, and study designs (10). This variability, particularly in outcome assessment, limits comparability and renders it difficult to draw definitive conclusions about the mechanisms underlying program effects (10).

Importantly, a central source of this heterogeneity lies in how MHL itself is assessed. Despite its prominent role as a core target of many prevention programs, there is currently no widely established instrument that adequately captures the multidimensional nature of the construct, meaning that conclusions about program effectiveness critically depend on how MHL is operationalized and measured. However, assessing MHL poses substantial methodological challenges, as the construct is latent, inherently multidimensional, and thus not directly observable. In practice, prevention studies therefore rely on a wide range of heterogeneous measurement approaches. Such assessments typically include self-developed, non-validated questionnaires (12, 17), questionnaires capturing only limited aspects of the MHL construct (18, 19), or vignette-based assessments (12, 17) that primarily focus on the recognition and stigma components of MHL. These approaches differ considerably in their conceptual focus and psychometric quality, and often fail to adequately capture the assumed multidimensional nature of MHL. As a result, findings across studies are difficult to compare and conclusions about program effectiveness remain limited. Moreover, inadequate measurement may lead to both under- and overestimations of intervention effects. Against this background, in order to meaningfully evaluate prevention programs targeting MHL, it is crucial to develop and validate a reliable and efficient questionnaire to assess the construct.

In view of the central role of gatekeepers’ MHL in prevention research, as well as the aforementioned lack of standardized and psychometrically sound assessment instruments, we developed the *Mental Health Literacy and Prevention Survey (mHELP)* as a brief, theory-informed instrument grounded in established conceptualizations of MHL and drawing on existing measures and current literature, with the participatory involvement of individuals with lived experience. The instrument was designed as an economical and time-efficient assessment of MHL in adults while adhering to established standards of scientific quality. The mHELP was previously administered within the context of an ongoing school-based study, and the present study therefore examines its psychometric properties in a sample of teachers, with the overall aim of evaluating whether the mHELP constitutes a suitable measurement instrument for MHL in adults. Specifically, the present study examined the questionnaire’s (1) factor structure, (2) reliability and internal consistency, and (3) construct validity. By providing a systematic psychometric evaluation, the study seeks to contribute to the standardization of MHL measurement and ultimately support future prevention research.

## 2 Materials and methods

### 2.1 Study design

The data for the present study were collected within the context of *tomoni.schools*, a nationwide school-based prevention study conducted in Germany using a long-term randomized controlled design. For the present psychometric evaluation of the mHELP, analyses were primarily based on baseline data. To examine the test–retest reliability of the instrument, we included six-month follow-up data from a subsample of control group participants. The study received ethical approval from the Ethics Committee of the German Psychological Society as well as the Ethics Committee of the Medical Faculty of the University of Würzburg, and was preregistered with the German Clinical Trials Register (DRKS; ID: DRKS00032839). Data collection was conducted online and included teachers from secondary schools across Germany. Prior to participation, all participants provided electronic informed consent. Participants then completed the self-report questionnaires. The follow-up questionnaire was administered 6 months after baseline following the same procedure. Participation was voluntary, and respondents were informed that they could withdraw from the study at any time without negative consequences. Data collection for the mHELP validation took place between December 2023 and November 2025.

### 2.2 Participants

The baseline sample consisted of *N* = 556 teachers from secondary schools in Germany who participated in the *tomoni.schools* study. Participants’ mean age was 43.1 years (SD = 9.27) and the majority identified as female (*n* = 478, 86.0%; male: *n* = 76, 13.7%; diverse: *n* = 2, 0.4%). Eligibility criteria for participation in the present analyses were employment as a teacher at a secondary school in Germany as well as registration for and provision of informed consent to participate in the *tomoni.schools* study. For the psychometric evaluation of the mHELP, only participants who completed the full mHELP questionnaire at the given measurement points were included in the analyses.

### 2.3 Measures

#### 2.3.1 mHELP

MHL was assessed using the mHELP, which was developed based on a comprehensive review of the literature on MHL and existing measurement instruments. Based on this framework, four core dimensions representing the subscales of the mHELP were defined *a priori*: (1) knowledge about mental illness, (2) stigma toward people with mental health difficulties, (3) confidence in one’s own helping behavior, and (4) self-reported helping behavior toward individuals experiencing mental health difficulties.

The initial item pool was developed by the research team based on the literature review, existing MHL-related instruments, and the intended use of the mHELP in prevention-oriented gatekeeper settings. Item content was partly adapted from established scales, particularly the Personal Depression Stigma Scale (20), and revised to align with broader definitions of MHL (9, 10, 21). Items adapted from existing English-language instruments were translated into German and conceptually adapted to the target context. The item pool was reviewed and revised by the authors with regard to conceptual fit, comprehensibility, redundancy, and feasibility for a brief survey instrument.

In addition to this literature-based development process, the mHELP was informed by a participatory approach involving close collaboration with individuals with lived experience of mental health problems, teachers, and tomoni mental health gGmbH. In a subsequent meeting with tomoni mental health gGmbH, the item pool was discussed in detail, including feedback from individuals with lived experience of mental health problems. During these exchanges, emotional understanding repeatedly emerged as an aspect considered highly relevant for sensitive and appropriate interactions with individuals experiencing mental health difficulties. Participants emphasized that, especially in prevention-oriented gatekeeper contexts, recognizing and responding to mental health concerns requires not only factual knowledge and confidence, but also an understanding of emotional distress and subjective emotional experiences.

Based on this practice-informed input, emotional understanding was included as a fifth component of the mHELP. It was conceptualized as an affective-interpersonal aspect that complements the four literature-based dimensions and is closely linked to the recognition of psychological distress, a core element of established MHL conceptualizations. At the same time, emotional understanding was distinguished from confidence, which refers to perceived self-efficacy in responding to mental health concerns, and from helping behavior, which refers to concrete supportive actions. In contrast, emotional understanding captures the extent to which respondents understand emotional distress and the subjective experience of mental health difficulties.

The mHELP consists of 19 items, which are rated on a 5-point Likert scale ranging from 1 (strongly disagree) to 5 (strongly agree). For all mHELP subscales and the total score, mean scores can be calculated, with higher values indicating higher levels of the respective construct.

#### 2.3.2 Additional measures

To examine construct validity, we administered additional established measures of MHL. The *Mental Health Knowledge Schedule* (MAKS; 19) is a six-item questionnaire designed to measure the knowledge component of MHL. The questionnaire has been validated in an Arabic version, with the authors reporting an internal consistency of Cronbach’s *α* = 0.56 (20).

Stigmatizing attitudes toward mental health problems were assessed using the *Questionnaire on Literacy and Stigma in Mental Health* (QLSM; 21). The original instrument comprises multiple subscales addressing knowledge and stigma across different domains related to mental health. For the purposes of this study, the stigma component (five items) was operationalized using the second factor of the questionnaire. For the original Portuguese version of the QLSM, internal consistency lay at Cronbach’s *α* = 0.82 (21).

Participants’ current mental health status was assessed using the *ICD-10-Symptom-Rating* [ISR; (22)], a standardized self-report instrument comprising 29 items designed to measure psychological symptom burden in accordance with ICD-10 diagnostic criteria. For the original German-language version of the scale, previous research has demonstrated excellent internal consistency, with a Cronbach’s alpha of *α* = 0.92 (23).

### 2.4 Statistical analyses

Prior to analysis, all negatively worded items of the mHELP were reverse-coded such that higher values corresponded to higher levels of MHL across all scales and subscales. In addition, all items of the QLSM were reverse-coded such that higher scores corresponded to higher levels of the construct.

To examine the factor structure of the mHELP, confirmatory factor analyses (CFA) were conducted using the lavaan package in R (24). A theoretically derived five-factor model was specified, representing the five dimensions. Models were estimated using robust maximum likelihood estimation (MLR) to account for potential deviations from multivariate normality. Missing data were handled using full information maximum likelihood (FIML). Based on these standardized factor loadings, explained variances, and modification indices, individual items were removed if they showed very low factor loadings or negligible explained variance. Model fit was evaluated using the Comparative Fit Index (CFI), Tucker–Lewis Index (TLI), Root Mean Square Error of Approximation (RMSEA, including 90% confidence intervals), and Standardized Root Mean Square Residual (SRMR).

Internal consistency of the mHELP was evaluated using reliability indices appropriate to the number of items for each respective scale. McDonald’s omega (ω) was calculated for the total scale score and for subscales consisting of five items, mean inter-item correlations were used for three-item subscales, and the Spearman–Brown coefficient was computed for two-item subscales.

Test–retest reliability was evaluated using data from a six-month follow-up assessment and Spearman’s rho correlation coefficients. In addition, split-half reliability was calculated for the mHELP total score. Items were alternately assigned to two parallel halves of the questionnaire, and Spearman’s rho was computed between the two split scores. Construct validity of the mHELP was examined by assessing Spearman’s rho correlations between mHELP subscale scores and the MAKS (convergent validity), the QLSM (convergent validity), and the ISR (discriminant validity).

## 3 Results

### 3.1 Descriptive statistics of mHELP

Descriptive statistics of the mHELP total score and subscales are presented in Table 1. Mean scores ranged from 2.65 to 4.02 across subscales, with the highest mean score observed for the Stigma subscale and the lowest for the Confidence subscale. The mean total mHELP score lay at 3.47 (SD = 0.56).

**Table 1:** Descriptive statistics for the mHELP total score and subscales (final 16-item version).

Scale	*M*	SD	Min	Max
Knowledge	3.51	0.74	1.00	5.00
Stigma	4.03	0.62	1.00	5.00
Confidence	2.65	0.86	1.00	5.00
Helping behavior	3.65	0.75	1.00	5.00
Emotional understanding	3.56	0.96	1.00	5.00
mHELP total score	3.47	0.54	1.50	4.88

Scores range from 1 to 5, with higher values indicating higher levels of the respective construct. Descriptive statistics are based on the final 16-item version of the mHELP after CFA.

### 3.2 Confirmatory factor analysis

Based on preliminary analyses, items 8, 13, and 17 were removed due to low explained variance and insufficient factor loadings for the CFA. The final five-factor model showed an acceptable model fit, *χ*^2^(94) = 385.22, *p* < 0.001, CFI = 0.903, TLI = 0.876, RMSEA = 0.075 (90% CI [0.067, 0.082]), and SRMR = 0.048.

All items loaded significantly on their intended latent factors (all *p*s < 0.001). Standardized factor loadings ranged from 0.25 to 0.89 (see Table 2). The Knowledge factor was indicated by five items, with standardized loadings between 0.61 and 0.75.

**Table 2:** Standardized factor loadings and explained variance (*R*^2^) for the final CFA model.

Factor	Item	Standardized loading	*R* ^2^
Knowledge	mHELP_01	0.70	0.49
	mHELP_02	0.75	0.57
	mHELP_03	0.61	0.37
	mHELP_04	0.67	0.45
	mHELP_05	0.74	0.54
Stigma	mHELP_06	0.74	0.55
	mHELP_07	0.45	0.20
	mHELP_09	0.25	0.06
Confidence	mHELP_10	0.77	0.59
	mHELP_11	0.55	0.30
	mHELP_12	0.75	0.57
Helping behavior	mHELP_14	0.69	0.48
	mHELP_15	0.73	0.53
	mHELP_16	0.62	0.39
Emotional understanding	mHELP_18	0.82	0.67
	mHELP_19	0.89	0.80

All factor loadings were statistically significant (*p* < 0.001). Loadings are standardized. *R*^2^ indicates the proportion of variance explained by the latent factor.

Latent factor correlations were positive and statistically significant (range from 0.35 to 0.76), indicating related but distinct constructs. The proportion of explained variance (*R*^2^) at the item level ranged from 0.06 to 0.80.

### 3.3 Internal consistency

Internal consistency was examined for the total mHELP score and for all subscales using reliability indices appropriate to the number of items per scale.

The total mHELP score showed high internal consistency (*ω* = 0.89). The Knowledge subscale (five items) also demonstrated good internal consistency (*ω* = 0.88). For the other subscales, internal consistency was lower (Stigma *ω* = 0.46, Confidence *ω* = 0.44, helping behavior *ω* = 0.74). For the three-item subscales, mean inter-item correlations were 0.21 for Stigma, 0.48 for Confidence, and 0.46 for helping behavior. The Emotional understanding subscale, consisting of two items, showed a strong inter-item correlation (*r* = 0.84), corresponding to a Spearman–Brown coefficient of 0.84. Reliability estimates for all scales are presented in Table 3.

**Table 3:** Internal consistency of the mHELP total score and subscales.

Scale	Items (*n*)	Reliability
Knowledge	5	*ω* = 0.88
Stigma	3	*ω* = 0.46/*r̄* = 0.21
Confidence	3	*ω* = 0.44/*r̄* = 0.48
Helping behavior	3	*ω* = 0.74/*r̄* = 0.46
Emotional understanding	2	SB = 0.84
mHELP total score	16	*ω* = 0.89

*ω*, McDonald’s omega; *r̄*, mean inter-item correlation; SB, Spearman–Brown coefficient. Mean inter-item correlations were reported for three-item subscales due to the limited number of items. For the two-item subscale, the Spearman–Brown coefficient was calculated.

### 3.4 Test–retest reliability

Test–retest reliability was examined using Spearman’s rank-order correlations in a subsample of participants who did not receive an intervention (*n* = 146). The mHELP total score showed moderate temporal stability (*ρ* = 0.64). Subscale correlations ranged from *ρ* = 0.45 to *ρ* = 0.57 (see Table 4).

**Table 4:** Test–retest reliability of the mHELP total score and subscales (Spearman’s *ρ*).

Scale	*ρ*
Knowledge	0.57
Stigma	0.53
Confidence	0.45
Helping behavior	0.51
Emotional understanding	0.46
mHELP total score	0.64

Test–retest reliability was examined using Spearman’s rank-order correlations.

### 3.5 Split-half reliability

Split-half reliability was additionally examined for the mHELP total score. The split-half coefficient was *r* = 0.89, indicating good internal consistency of the overall questionnaire.

### 3.6 Construct validity

The Knowledge subscale of the mHELP (see Table 5) showed a moderate positive correlation with the MAKS (*ρ* = 0.38, *p* < 0.001). The Stigma subscale showed a small positive correlation with the QLSM (*ρ* = 0.25, *p* < 0.001).

**Table 5:** Spearman correlations between mHELP subscales and established measures.

Scale	MAKS	QLSM	ISR
Knowledge	0.38^***^	0.18^***^	0.07
Stigma	0.20^***^	0.25^***^	−0.04
Confidence	0.29^***^	0.11^**^	−0.03
Helping behavior	0.28^***^	0.19^***^	−0.07
Emotional understanding	0.22^***^	0.05	0.29^***^
mHELP total score	0.40^*^	0.22^*^	0.06

Spearman’s rho correlations are reported. MAKS, Mental Health Knowledge Schedule; QLSM, Questionnaire Literacy and Stigma in Mental Health; ISR, psychological distress.^*^p < 0.05, ^**^p < 0.01, ^***^p < 0.001 (one-tailed).

Correlations between the mHELP total score and both established measures were smaller but statistically significant. Associations between mHELP scores and psychological distress as measured by the ISR were generally small. The mHELP Emotional understanding subscale showed a small positive correlation with ISR scores (*ρ* = 0.29, *p* < 0.001), whereas correlations with the remaining subscales and the total score were weak or non-significant.

## 4 Discussion

The present study aimed to develop and validate the mHELP, a multidimensional instrument designed to assess MHL among gatekeepers within prevention research. Overall, the findings provide initial support for the mHELP as a brief measure of MHL, with stronger psychometric evidence for the total score than for some individual subscales. Confirmatory factor analyses indicated an acceptable, albeit not unequivocal, fit for the proposed five-factor structure, as several fit indices were acceptable whereas the TLI remained below conventional thresholds. The refined 16-item version retained the theoretically intended domains—knowledge, stigma, confidence in one’s own helping behavior, self-reported helping behavior, and emotional understanding— and represents a more parsimonious and practically applicable instrument, although not all subscales showed equally robust psychometric properties. The removal of three items was theoretically and empirically justified and did not compromise the conceptual breadth of the scale, but rather improved its structural coherence and applicability. Although item 9 showed low explained variance, it was retained because its removal did not improve model fit and the item was considered conceptually relevant for capturing stigmatizing attitudes. Nevertheless, the weak performance of this item should be considered when interpreting the Stigma subscale and further underscores the need for future refinement of this subscale.

With regard to reliability, the total score as well as the Knowledge, Self-reported helping behavior, and Emotional understanding subscales showed satisfactory to good reliability. Psychometric findings for the Confidence subscale were mixed, with acceptable item homogeneity but low omega, indicating that it should not yet be interpreted as a fully reliable independent subscale. In contrast, the Stigma subscale demonstrated insufficient internal consistency and appears to require further item refinement before separate interpretation can be recommended. Overall, the total score currently represents the most robust indicator of MHL.

Split-half reliability of the total score was high, and test–retest analysis over a six-month interval indicated moderate temporal stability. The latter finding may partly reflect the fact that MHL is not a fixed trait but rather a modifiable prevention target that can change over time. The relatively long retest interval likely also contributed to lower stability estimates compared with validation studies using shorter intervals.

Initial evidence of construct validity was provided by theoretically meaningful associations with established measures. As expected, the mHELP Knowledge subscale showed the strongest associations with the MAKS (19), and the Stigma subscale was most strongly related to the QLSM, providing initial support for convergent validity. However, some associations were small in magnitude, and both established measures also showed significant correlations with the remaining mHELP subscales. This pattern is not unexpected given the moderate to strong intercorrelations among the mHELP subscales themselves, and suggests that the questionnaire captures related but non-independent facets of a broader MHL construct rather than strictly separable dimensions. The finding of weak or absent associations with the ICD-10-Symptom-Rating (23) for most subscales provides preliminary support for discriminant validity of the instrument.

The current study contributes to MHL research by providing initial psychometric evidence for a brief questionnaire aligned with established definitions of MHL (based on 9–11). By seeking to capture multiple relevant facets within a single instrument, the mHELP may complement existing measures that often focus on isolated components such as knowledge or stigma. Notably, the inclusion of emotional understanding as a distinct subscale represents a novel contribution. This dimension emerged from participatory work involving individuals with lived experience of mental health problems, and the preliminary empirical findings suggest that this practice-informed dimension warrants further investigation. Emotional understanding may therefore be an important and previously underrepresented facet of MHL that warrants stronger consideration in future conceptualizations of the construct. Conceptually, emotional understanding is related to empathy, a construct that is well established in psychological research. Previous studies indicate associations between MHL and trait empathy (25), and there is initial evidence linking trait empathy to helping tendencies (26). However, given that the mHELP is intended for use in prevention and intervention contexts, the emotional understanding subscale focuses less on empathy as a stable dispositional characteristic and more on state-like empathic components. Such state-like components of empathy have been described as context-dependent and potentially modifiable, for instance through knowledge, situational appraisals, and other contextual factors (27). To our knowledge, however, no study has yet directly examined how these state-like components of empathy relate to MHL. Future research should therefore investigate more specifically how emotional understanding relates to both trait and state empathy, and whether it can be improved through MHL training.

Compared with existing instruments, including questionnaires or vignette-based approaches, the mHELP was designed to provide a broader and more integrated assessment of MHL. Measures such as the MAKS or the vignettes used by Jorm (11) capture relevant aspects of MHL but do not reflect the multidimensional structure of the construct. Moreover, several established instruments exhibit pronounced ceiling effects and low variance (see Supplementary material), limiting their sensitivity in prevention and intervention research. The mHELP demonstrated sufficient variability across subscales, suggesting that it may be better suited to detect changes over time, including intervention-related effects.

A further strength of the mHELP is the inclusion of a subscale assessing self-reported helping behavior. To date, behavioral components of MHL have rarely been measured directly, despite their central relevance for preventive action by gatekeepers. The mHELP allows this dimension to be assessed in a standardized self-report format. However, items 15 and 16 should be interpreted as indicators of intended rather than actual helping behavior and may thus overestimate actual behavior due to the well-established discrepancy between self-reported and actual behavior as well as the intention-behavior gap (28, 29). Future research could therefore complement this approach by incorporating behavioral indicators, external ratings, scenario-based assessments, standardized role-play assessments, or virtual reality-based simulations to further strengthen the assessment of helping behavior and the validity evidence for the mHELP.

The mHELP was intentionally designed as a brief and economical instrument, reflecting the practical requirements of prevention research and applied settings. Its structure renders it suitable for use across the three main levels of prevention (universal, selective, and indicated prevention) (8). Given the characteristics of the present sample, the mHELP appears particularly well suited for school-based and educational prevention contexts. At the same time, its conceptual breadth suggests potential applicability beyond the school context, including wider gatekeeper populations such as parents, social workers, or coaches. However, its use in these groups requires further empirical validation. With its low respondent burden and flexible application, the instrument is not only appropriate for efficacy studies but also highly relevant for implementation research and health services research, where feasibility, scalability, and cost-effectiveness are key considerations. Future research should therefore examine the mHELP in population-based samples and pursue cross-cultural validation, including of an English-language version, to further enhance its generalizability and international relevance.

### 4.1 Limitations

The findings of this study should be interpreted in light of several limitations. First, the sample consisted exclusively of teachers in Germany and was predominantly female. While this focus is highly relevant for prevention research in school and other educational settings, the current findings primarily support the use of the mHELP among teachers in the German educational context. Generalizability to other gatekeeper groups, such as parents, youth workers, counselors, social workers, community volunteers, or coaches, requires further empirical investigation.

Second, all constructs were assessed using self-report measures, which may be subject to social desirability bias, self-perception bias, and common method bias. This may be particularly relevant for the assessment of stigma, confidence, and helping behavior, as self-reported attitudes, perceived self-efficacy, and intended helping behavior may not fully reflect actual behavior in real-life situations.

Third, the economical design resulted in relatively short subscales, which may have limited the reliability of some domain scores, particularly the Stigma subscale. Accordingly, the total score currently appears to provide the most robust overall indicator, while selected subscales, particularly Stigma and Confidence, require further refinement before strong conclusions can be drawn from separate subscale scores.

Fourth, item removal, model refinement, and evaluation of the final CFA model were conducted using the same dataset, which may have increased the risk of overfitting and may have led to an overly optimistic estimation of model fit. The proposed five-factor structure therefore requires cross-validation in an independent sample before the factorial validity of the refined mHELP can be considered firmly established.

Fifth, although test–retest reliability was assessed over a relatively long interval, longitudinal measurement invariance and sensitivity to change were not examined. Future studies should address these aspects to establish the stability of the measure and its suitability for intervention research.

Sixth, items 15 and 16 assess self-reported helping tendencies rather than actual helping behavior. As a result, responses may overestimate actual helping behavior due to the well-established discrepancy between self-reported and actual behavior as well as the intention-behavior gap (28, 29). Furthermore, responses to these items may partly reflect teachers’ perceptions of their professional role and responsibility in addressing students’ mental health concerns rather than solely their helping tendencies or perceived competence. At the same time, assessing actual helping behavior in educational settings is challenging because opportunities to engage in helping behavior vary substantially across teachers and may not occur within a given assessment period. Consequently, self-reported helping tendencies may represent a pragmatic approach for prevention and intervention studies using pre–post designs. Future studies should nevertheless examine more closely the extent to which these items capture helping tendencies, self-efficacy, and role-related beliefs.

Seventh, the mHELP primarily focuses on the detection of and response to mental health problems. It does not assess whether teachers are able to recognize students’ own mental health promotion skills or competencies that support positive mental health, as reflected, for example, in dual-continuum models of mental health (30). Future extensions of the mHELP could therefore consider including items that capture teachers’ ability to identify not only mental health difficulties, but also students’ strengths and competencies related to positive mental health.

Finally, normative reference values are not currently available, and the interpretation of individual scores is thus limited to relative comparisons within study samples. The development of normative data for different populations would enhance the applicability of the mHELP for screening, evaluation, and applied prevention contexts.

### 4.2 Conclusion and future directions

The present study provides initial psychometric evidence for the mHELP as a brief instrument for assessing mental health literacy (MHL) among gatekeepers in prevention research. The mHELP conceptualizes MHL as a multidimensional construct encompassing knowledge, stigma, confidence in one’s own helping behavior, self-reported helping behavior, and emotional understanding. By integrating theoretical foundations with participatory scale development and empirical validation, the mHELP constitutes a concise and practical tool suitable for prevention and implementation contexts. Its brief and economical format facilitates application in settings with limited time and resources. While the total score currently appears to be the most robust indicator, future refinement of selected subscales - particularly the Stigma and Confidence subscales - may further enhance the multidimensional utility of the instrument. Moreover, normative data based on population-based samples are needed to improve interpretability and facilitate comparisons across studies and settings. Validation in more diverse samples beyond teachers and the German educational context will be essential to establish broader applicability. Finally, the development and validation of a child and adolescent version of the mHELP represents an important next step, given the relevance of MHL across the lifespan. Together, these directions will further strengthen the mHELP as a versatile instrument for research and practice in mental health prevention.

## Data Availability

The raw data supporting the conclusions of this article will be made available by the authors, without undue reservation.

## Mental Health Literacy and Prevention Survey (mHELP)

**Table:**

German version Ich kenne Anzeichen psychischer Erkrankungen. Ich kann Fragen zu psychischen Erkrankungen von Freund*innen/Familienmitgliedern/Bekannten beantworten. Ich kenne Hilfsangebote und Anlaufstellen für Menschen, die Anzeichen einer psychischen Erkrankung zeigen. Ich weiß, wo ich zuverlässige Informationen zum Thema psychische Gesundheit erhalte. Ich kenne einige Strategien (Dos and Don’ts) im Umgang mit Menschen, die Anzeichen einer psychischen Erkrankung zeigen. Ich stehe Menschen, die psychisch erkrankt sind, offen gegenüber. Ich würde jemanden, von dem ich weiß, dass er psychisch erkrankt ist, nicht für mein Team auswählen. Menschen mit einer psychischen Erkrankung könnten allein davon loskommen, wenn sie es wollten.^†^ Wenn ich eine psychische Erkrankung hätte, würde ich niemandem davon erzählen. Ich fühle mich sicher darin, Menschen, die Anzeichen einer psychischen Erkrankung zeigen, anzusprechen. Ich habe Angst davor, im Umgang mit Menschen, die Anzeichen einer psychischen Erkrankung zeigen, etwas falsch zu machen. Ich fühle mich sicher darin, Menschen, die Anzeichen einer psychischen Erkrankung zeigen, helfen zu können. Ich fühle mich sicher darin, bei einer eigenen psychischen Belastung zu wissen, was ich tun kann.^†^ Ich kann Menschen mit Anzeichen einer psychischen Erkrankung zeigen, dass ich ansprechbar bin, wenn sie darüber reden wollen. Ich spreche Menschen (auch wiederholt) darauf an, wenn mir Anzeichen einer psychischen Erkrankung auffallen und ich denke, dass es ihnen nicht gut geht. Ich suche mit Menschen, die Anzeichen einer psychischen Erkrankung zeigen, gemeinsam nach Unterstützung (z. B. Hilfsangebote wie Beratungsstellen oder Psychotherapie). Ich suche mir Hilfe, wenn ich bei mir selbst Anzeichen einer psy. Erkrankung erkenne.^†^ Ich kann nachvollziehen, wie sich die Belastung durch eine psychische Erkrankung für betroffene Personen anfühlt. Ich kann mich in Menschen, die Anzeichen einer psychischen Erkrankung zeigen, hineinversetzen.
English translation I am familiar with signs of mental disorders. I am able to answer questions about mental disorders from friends, family members, or acquaintances. I am familiar with support services and points of contact for people who show signs of a mental disorder. I know where to find reliable information on the topic of mental health. I am familiar with strategies (dos and don’ts) for interacting with people who show signs of a mental disorder. I am open-minded toward people with mental disorders. I would not select someone for my team if I knew that they had a mental disorder. People with a mental disorder could overcome it on their own if they really wanted to.^†^ If I had a mental disorder, I would not tell anyone about it. I feel confident in approaching people who show signs of a mental disorder. I am afraid of doing something wrong when interacting with people who show signs of a mental disorder. I feel confident in my ability to help people who show signs of a mental disorder. I feel confident that I would know what to do if I experienced psychological distress myself.^†^ I am able to show people who display signs of a mental disorder that I am approachable if they want to talk about it. I approach people (even repeatedly) when I notice signs of a mental disorder and think that they are not doing well. I help people who show signs of a mental disorder to look for sources of support (e.g., counseling services or psychotherapy). I seek help when I notice signs of a mental disorder in myself.^†^ I can understand what the burden of a mental disorder feels like for those affected. I am able to empathize with people who show signs of a mental disorder.

^†^Indicated items were removed following the confirmatory factor analysis (CFA). All items were rated on a 5-point Likert scale ranging from 1 = strongly disagree to 5 = strongly agree.
