# Supplementary material for: The Mental Health Literacy and Prevention Survey (mHELP): a brief measure for assessing gatekeepers’ mental health literacy in prevention research
Source: Front Public Health. 2026 Aug 18;14:1868366. doi: 10.3389/fpubh.2026.1868366 (PMC13531427; doi:10.3389/fpubh.2026.1868366)
Supplement: Supplementary file 1 [file Table_1.pdf]

Supplementary Material

Scale	*M*	*MD*	*SD*	*Min*	*Max*
Knowledge	3.50	3.60	0.75	0.00	5.00
Stigma	4.02	4.25	0.65	0.00	5.00
Confidence	2.65	2.75	0.87	0.00	5.00
Helping Behavior	3.64	3.75	0.76	0.00	5.00
Emotional Understanding	3.56	4.00	0.97	0.00	5.00
mHELP total score	3.47	3.26	0.56	0.00	4.88
QLSM	4.58	4.4	0.43	2.00	5.00
MAKS	4.11	3.83	0.50	0.00	5.00

Note. Scores range from 1 to 5, with higher values indicating higher levels of the respective construct.
Mhelp: Descriptive statistics are based on the final 16-item version of the mHELP after CFA.
